# Cost-effectiveness analysis of mammography screening for early detection of breast cancer in Nigeria

**DOI:** 10.1371/journal.pone.0351492

**Published:** 2026-06-12

**Authors:** Ifeoma Jovita Nduka, Charles Ebuka Okafor, Obinna Ikechukwu Ekwunife

**Affiliations:** 1 Department of Clinical Pharmacy and Pharmacy Management, Faculty of Pharmaceutical Sciences, Nnamdi Azikiwe University, Awka, Nigeria; 2 Centre for Health Services Research, Faculty of Health, Medicine and Behavioural Sciences, The University of Queensland, Brisbane, Qld Australia; 3 Division of Population Health, Department of Medicine, Jacobs School of Medicine and Biomedical Sciences, University at Buffalo, Buffalo, New York, United States of America; Local Health Authority Caserta: Azienda Sanitaria Locale Caserta, ITALY

## Abstract

Mammography still remains the gold standard for breast cancer screening, considering its impact on breast cancer mortality. However, it has a relatively low utilization rate in Nigeria. Although the National Strategic Cancer Control Plan (NSCCP) has a goal of making screening services and early detection of cancer available for all Nigerians, there is currently no national breast cancer screening program implemented in Nigeria. The modelling study aimed to evaluate the cost-effectiveness of mammography screening from the healthcare provider’s perspective and to determine the appropriate screening interval for Nigerian women, aiming to enhance the efficiency and effectiveness of breast cancer detection programs. A state-transition Markov model was adapted to simulate annual and biennial mammography, breast cancer diagnosis, and treatment in a cohort of cancer-free Nigerian women aged 40 years and followed them for a lifetime. The study was conducted from the healthcare provider’s perspective. Disability-adjusted life year (DALY) averted, representing the health outcomes, was used to estimate the incremental cost-effectiveness ratio (ICER). Costs and outcomes were discounted at an annual rate of 5%. Annual mammography screening costs US$238.60, averted a DALY of 1.060, and was the most cost-effective intervention with an ICER of US$207.24 (95% CI US$213.31 – US$216.88)/DALY averted, which was below the willingness-to-pay threshold of $1074. Mammography screening strategies were estimated to be cost-effective from the healthcare payer’s perspective under the model assumptions. Annual screening showed the most favorable cost-effectiveness profile among the strategies evaluated, but this finding is model-dependent and should be interpreted as comparative economic evidence rather than a definitive screening recommendation. These results can inform future research, policy discussions, and consideration of sustainable financing for breast cancer screening in Nigeria.

## Introduction

With a global estimate of 2.3 million new cases (23.8%), female breast cancer is the most commonly diagnosed cancer [1], accounting for 15.4% of cancer deaths with considerably higher death rates in low- and middle-income countries (LMICs) [1,2]. In Nigeria, breast cancer remains the most prevalent cancer, accounting for 40.5% of new cancer cases, and the leading cause of cancer-related death (35.0%) [2].

Mammography is considered the gold standard for breast cancer screening due to its proven ability in the timely detection of breast cancer in asymptomatic individuals and reduction in breast cancer-related deaths by 15–56% [3–5]. However, its effectiveness is dependent on the adherence rate. As such adherence to routine mammography screening is crucial [5,6]. As early diagnosis of breast cancer coupled with comprehensive treatment results in improved prognosis of the disease and a reduction in mortality rate, its need cannot be overemphasized [7]. In Nigeria, early diagnosis of breast cancer encounters numerous challenges resulting in late presentation. These challenges range from poor symptom recognition, inadequate knowledge, poor healthcare system, financial constraints, fear of diagnosis, and reliance on traditional medicine [8,9].

Currently, there is no national breast cancer mammography screening program in Nigeria, unlike in some advanced regions (e.g., USA), and developing nations (e.g., Colombia) [4,10–17] where population-based mammography screening has been widely implemented [5]. Most of the national guidelines recommend biennial screening for 50–69 year-old women with an average risk of breast cancer development [18], while a few countries implemented different screening policies, such as in the UK, where triennial screening for the same age groups is recommended [19]. These countries provide mammography screening at no cost or a heavily subsidized cost, as the expense is typically funded by the government and health insurance, resulting in minimal or no out-of-pocket payments [20–22]. A Nigerian study indicated that over 80% of Nigerians have access to diagnostic imaging services, including mammography, for the timely detection of breast cancer [23]. Notwithstanding, the mammography utilization rate remains low in Nigeria, even among healthcare workers [24–26]. This might be due to various reasons, including out-of-pocket expenses associated with the screening [26–28]. Hence, a nationwide mammography screening initiative, with minimal or no out-of-pocket expenses, could reduce the instances of late-stage cancer diagnoses, lower the likelihood of breast cancer-related deaths, and address current disparities in cancer care in Nigeria [29].

In response to the cancer menace, the Federal Government of Nigeria established the National Cancer Control Programme. One of the objectives of the National Cancer Control Plan (NCCP) 2018–2022 is to ensure that all Nigerians have access to screening services and early cancer detection [30]. Some of the goals, like increased access to cancer diagnostics and treatment, and effective cancer awareness and sensitization campaigns, were achieved and are progressing, respectively. Meanwhile, other goals, such as the integration of primary prevention/cancer screening into primary healthcare delivery, have not been implemented [31]. Following the elapse of the timeline for the 2018–2022 plan, the National Strategic Cancer Control Plan 2023-2027 (NSCCP) was developed, having its top priority area of action as the prevention of cancers [31]. Hence, NSCCP aims to achieve the screening of more than 50% of the eligible population by 2027, through the establishment of nationwide routine screening for cancers, including breast cancer [31].

Nigeria operates a diverse healthcare system with private and public sectors, modern and traditional systems providing health care [32,33]. The public healthcare sector is the responsibility of the three tiers of government; the federal, state, and local government. The local governments are responsible for primary health care (PHC) services, whereas the state and federal governments carter for secondary and tertiary level care, respectively [32]. The thriving private health sector renders about 60% of the health care services through 30% of the country’s conventional health facilities, such as not-for-profit services provided by faith-based and non-governmental organizations; and private-for-profit providers. Other private health sector includes traditional medicine providers, patent and proprietary medicine vendors (PPMVs), and complementary and alternative health practitioners [32]. Reviews showed that mammography screening is mostly unstructured in Nigeria, regarding the invitation mode, age of participants, and frequency of screening, with fewer than 40% of tertiary health institutions having functional mammography units [28]. Hence, other non-governmental organizations and multinational organizations have been involved in the provision of mammographic breast screening in Nigeria.

The Nigerian healthcare system practices a liberal healthcare system model, [34] in which payment for health expenditures are mainly made by patients through out-of-pocket (OOP) payments [5]. This OOP is responsible for about 69% of overall healthcare spending [5,35,36]. The level of public funding for healthcare is minimal, captured by the small fraction of public health expenditure to the total government spending in the Nigerian budget [5]. The reduction of the federal budget for health from 5.75% in 2023 to 5.15% in 2024, and then further to 4.99% in 2025 validates the minimal public funding for healthcare [5,37]. The National Health Insurance Authority (NHIA), a social health insurance program established in 2005 with an emphasis on targeted health funding and private healthcare delivery, could ideally support public initiatives like mammography. Conversely, it is not universally accessible, as fewer than 10% of the population is subscribed to it [38–40], and it does not provide adequate financial protection for breast screening, as coverage is not universal across all plans [25,41]. The nation seeks to reverse the above by signing into law the new NHIA Act of 2022, which aims to secure mandatory health insurance for every Nigerian [39]. As the National Strategic Cancer Control Plan (NSCCP) works toward establishing sustainable financing mechanisms to improve access to cancer screening, evaluating the value for money of a national mammography screening programme in Nigeria is essential to support decision making. Although a randomized controlled trial–based economic evaluation would be ideal, such studies are not currently feasible in Nigeria due to substantial data and resource constraints. In this context, a robust modelling approach provides a feasible and complementary source of evidence for comparing alternative screening scenarios under limited data conditions. A review of the existing literature indicates that no prior study has evaluated the cost-effectiveness of mammography screening in Nigeria. Therefore, this study assessed the cost-effectiveness of alternative mammography screening scenarios among Nigerian women within a data-constrained setting.

## Methods

A model that included both the epidemiological and cost aspects of breast cancer was designed to estimate the cost-effectiveness of a screening program for breast cancer. Also, sensitivity analyses were conducted to assess uncertainty about the model parameters.

### Markov model structure

A state-transition Markov model was adapted to inform the decision [42]. The model predicted the lifetime costs and disability-adjusted life years (DALYs) of screening versus no screening for Nigerian women with no prior history of breast cancer, from age 40 years for a time horizon of 45 years. The cohort of 1,035,063 asymptomatic women aged 40 years was based on the 2019 population of Nigeria [43]. The epidemiological variability of breast cancer and its sequels on three age groups, 40–49, 50–59, and 60–69 years, was applied in the model. We used a no screening scenario as the base case, which was compared to the scenarios of annual and biennial mammography screening, assuming a 100% participation rate. Alternatively, a scenario analysis of 50% participation rate was evaluated.

**Fig 1:**
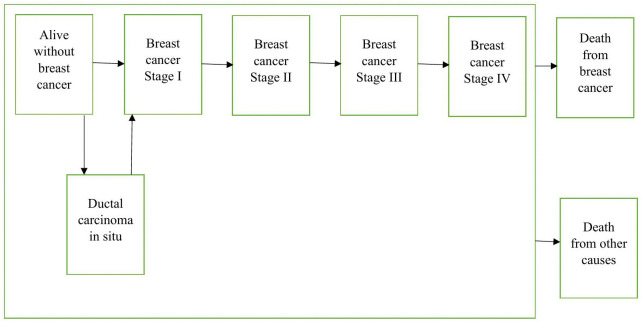
Natural history model for breast cancer progression adapted from Sun et al., 2018.

Fig 1 illustrates the health states and the potential transitions between them.

A cohort of 40-year-old cancer-free women was tracked for a lifetime by the model. These women may remain free of breast cancer, develop ductal carcinoma in situ (DCIS), or invasive breast cancer. Women with ductal carcinoma in situ have a higher risk of developing invasive breast cancer (relative risk: 2.02) [42]. Stage I patients can progress to stage II, stage III, and stage IV. All women can die from causes other than breast cancer during disease progression, but only patients at stage IV can die from breast cancer [42]. The state transition probabilities were based on incidence rates for breast cancer, breast cancer mortality, and mortality for other causes, based on available evidence in the literature. We presumed that all breast cancer patients underwent prompt and adequate treatment [44].

### Epidemiological and clinical data

Key parameters are summarized in Table 1. We used age-specific breast cancer incidence rates from online data from the Ibadan Cancer Registry [45]. Transition probabilities between health states in the Markov model were applied from published literature [46,47]. Stage-specific mortality probabilities were derived from a study [48]. The probability of death from other causes was defined as the difference between age-specific mortality probabilities from all causes and those from breast cancer. The all-cause mortality probability by age group and breast cancer mortality rate by age were obtained from Nigerian life tables for women in 2019 and the database of the Institute for Health Metrics and Evaluation (IHME), respectively [49,50].

**Table 1:** Parameter Values for Modelling the Cost-effectiveness of Breast Cancer Screening in Nigeria.

Variables	Baseline	Minimum	Maximum	Distribution	Reference/Source
**Disease state progression transition probabilities**					
**Age-specific incidence, years**					
40–49	0.001048	0.000786	0.001310	Beta	[45]
50-59	0.001292	0.000969	0.001615	Beta	[45]
60-69	0.001002	0.000752	0.001253	Beta	[45]
Ratio of DCIS incidence to invasive breast cancer incidence	0.12	–	–		[42]
RR of invasive breast cancer from DCIS	2.02	–	–	–	[42]
**Progression rate**					
Stage 0 – I	0.2	0.169940	0.226587	–	
Stage I – Stage II	0.06	0.043677	0.072794	–	[46]
Stage II – Stage III	0.11	0.078124	0.130207	–	[46]
Stage III – Stage IV	0.15	0.104469	0.174115	–	[46]
Stage IV – Death	0.23	–	–	–	[47]
**All-cause mortality rate**					
40–49	0.004996	0.003813	0.006798		[50]
50–59	0.009370	0.006887	0.012548		[50]
60–69	0.021218	0.016158	0.026680		[50]
**Breast cancer mortality rate**					
40–49	0.000285	0.000189	0.000430		[50]
50–59	0.000633	0.000423	0.000913		[50]
60–69	0.001023	0.000715	0.001395		[50]
**Annual fatality rate after treatment/Stage-specific mortality rate**					
Stage I	0.006	0.004010	0.008660	Beta	[48]
Stage II	0.042	0.028040	0.060600	Beta	[48]
Stage III	0.093	0.062090	0.134190	Beta	[48]
Stage IV	0.275	0.183610	0.396810	Beta	[48]
**Effectiveness of screening**					
Sensitivity	0.747	0.414	0.971	Log normal	[51–53]
Specificity	0.834	0.554	0.980	Log normal	[51–53]
**Disability weights**					
DCIS	0.049	0.031	0.072	Beta	[50]
Stage I	0.083	0.052	0.124	Beta	[50]
Stage II	0.288	0.193	0.399	Beta	[50]
Stage III	0.451	0.307	0.600	Beta	[50]
Stage IV	0.54	0.377	0.687	Beta	[50]
**Costs in Naira (USD)**					
Screening	3000 (7.50)	5.25	9.75	Gamma	Medical record
Diagnosis	120500 (301.25)	210.88	391.63	Gamma	Medical record
**Treatment costs in Naira (USD)**					
DCIS	1040525 (2601.31)	1820.92	3381.70	Gamma	Medical record
Stage I	1040525 (2601.31) **or** 1128295 (2820.74)	1820.92 **or** 1974.52	3381.70 **or** 3666.96	Gamma	Medical record
Stage II	1040525 (2601.31) **or** 1128295 (2820.74)	1820.92 **or** 1974.52	3381.70 **or** 3666.96	Gamma	Medical record
Stage III	1028415 (2571.04)	1799.73	3342.35	Gamma	Medical record
Stage IV	1068795 (2671.99)	1870.39	3473.59	Gamma	Medical record

### Costs

The cost was projected from the healthcare payer’s perspective [54]. Only the direct medical costs were included, which comprised the cost of mammography screening, diagnostic workup, and breast cancer treatment. These costs (Table 2) and the Breast Cancer Management Protocol [55] were derived from the University of Nigeria Teaching Hospital, Ituku-Ozalla, Enugu State.

**Table 2:** Patient-Level Resource Use Patterns for Breast Cancer Interventions.

Parameter	Resources^a^	Costs in Naira (USD)	No of outpatient visit^b^/length of hospitalization (days)^c^
**Screening**	Bilateral mammography	3000 (7.50)	1/NA
**Diagnosis**	Bilateral mammography Trucut biopsy (Core biopsy) Immunohistochemical analysis Chest x-ray Abdominopelvic ultrasound Bone scan/x-ray Full blood count Retroviral status Liver function test Serum urea creatinine Alkaline phosphatase, serum calcium Electrocardiography (ECG) 2-D Echocardiography	3000 (7.50) 30000 (75.00) 24000 (60.00) 3500 (8.75) 4000 (10) 7800 (19.50) 2000 (5.00) 700 (1.75) 7000 (17.50) 8500 (21.25) 4500 (11.25) 5000 (12.50) 17500 (43.75)	1/NA
**Stage 0 (DCIS) treatment**	Breast-conserving surgery (BCS) Whole breast radiotherapy (WBRT) Adjuvant chemotherapy Endocrine therapy	49930 (124.83) 470000 (1175.00) 301920 (754.80) 209875 (524.69)	½
**Stage I treatment**	BCS plus WBRT Or Mastectomy plus Chest wall radiotherapy Adjuvant Chemotherapy Endocrine therapy	49930 (124.83) 470000 (1175.00) 137700 (344.25) 470000 (1175.00) 301920 (754.80) 209875 (524.69)	1/2
**Stage II treatment**	BCS plus WBRT Or Mastectomy Chest wall radiotherapy Adjuvant Chemotherapy Endocrine therapy	49930 (124.83) 470000 (1175.00) 137700 (344.25) 470000 (1175.00) 301920 (754.80) 209875 (524.69)	½
**Stage III treatment**	Neoadjuvant chemotherapy Mastectomy with axillary dissection Radiotherapy Endocrine therapy Bisphosphonate therapy	123840 (309.60) 137700 (344.25) 470000 (1175.00) 209875 (524.69) 78200 (195.50)	½
**Stage IV treatment**	Adjuvant Chemotherapy Endocrine therapy Bisphosphonate therapy Palliative radiotherapy	301920 (754.80) 209875 (524.69) 78200 (195.50) 470000 (1175.00)	1/2

The average conversion factor of 0.0025 per dollar for June 2023

a Based on the University of Nigeria Teaching Hospital Enugu Breast Cancer Management Protocol

b Outpateint visit cost – 3000 (7.50)

^c^Hospitalization cost – 2900 (7.25)

For breast cancer treatment, the following considerations were made. Endocrine therapy consisted of 20 mg of tamoxifen per day for 5 years. Radiotherapy included a standard dose of 50Gy given in 25 fractions of 2 Gy on an outpatient basis in all stages of breast cancer. The adjuvant chemotherapy combination consisted of four 21-day cycles of doxorubicin (60 mg/m^2^) and cyclophosphamide (600 mg/m^2^), followed by weekly paclitaxel (80 mg/m^2^) for 12 weeks. The neoadjuvant chemotherapy combination consisted of eight, 21-day cycles of doxorubicin (60 mg/m^2^) and cyclophosphamide (600 mg/m^2^). Bisphosphonate therapy consisted of zoledronic acid (4 mg), 21-day cycles for 1 year (approximately 17 doses). An average body surface area of 1.84m^2^, derived from the breast cancer patients’ treatment folders, was used to calculate the chemotherapy dose. All costs were adjusted to 2021 values. These costs were discounted at an annual rate of 5%.

### Effectiveness of screening

The sensitivity and specificity of mammography screening were obtained from published Nigerian studies [51–53]. We varied the estimates in the sensitivity analyses to test for the effect of any variation in diagnostic performance.

### Health outcome

The disability-adjusted life years (DALYs) was used to represent the health outcome. [54]. The DALY is a health outcome measure that has two main components: the duration of a lifetime lost due to premature death (years of life lost (YLL)), and the reduction in quality of life due to a disability (years of life with a disability (YLD)). The primary scale is 0 (perfect health with no disability) to 1 (dead). The recent Global Burden of Disease 2019 study was used to calculate the DALY, utilizing updated disability weights [54,56]. The DALYs per patient was determined by computing the DALYs for each cycle, adding it over the model time horizon, and obtaining the average [54]. We discounted future benefits at 5%. Also, mortality rate was employed as a secondary health outcome.

### Model validation

The Markov model was validated internally and externally. The model’s structural validity, an indicator of how well a model reflects reality, was determined through descriptive validity to assess whether the degree of simplification used in the model structure still adequately represents the natural history of breast cancer and pathways of care. Descriptive validation was performed with the experts (oncology surgeons) who were consulted to provide insights into the natural course of breast cancer and its management in Nigeria. Descriptive validity was carried out on the ability of the model structure to incorporate the assumptions about the development and current care of breast cancer into a simplified structure, to answer the decision problem.

Technical validity was assessed to determine whether the Markov model was appropriately designed to produce the intended outputs from the specified inputs. The Markov model was checked for programming errors, data entry errors, and logical inconsistencies in the model specification.

Predictive validity was conducted to assess whether our model outputs sufficiently represent outputs from alternative sources. This was assessed by comparing the outputs of total DALY, total YLD, and total YLL by age group with the 2019 Global Burden of Disease data.

### Analysis

The treatment course/cost for each intervention was calculated by summing up the cost components [54]. For each intervention cycle, the cost was obtained by adding the number of breast cancer patients and multiplying it by the cost of management, followed by applying a discount. The cost for the 45 cycles was totaled and averaged to determine the cost per patient (standard cost). The standard cost of each intervention was subsequently used to conduct the univariate sensitivity analysis and probabilistic sensitivity analysis (PSA) [54].

We obtained the DALY by adding the YLD and YLL for each cycle. [54]. YLD was calculated by multiplying the number of cases by the disability weight and then discounted. The disability weights for patients at DCIS, stage I, II, III, and IV originated from the global burden of disease (GBD) study [56]. YLL was calculated by multiplying the number of deaths due to breast cancer by the life expectancy at the age of death. The standard life expectancy by age was obtained from the World Health Organization database [49]. The standard DALYs, utilized in the PSA, was obtained by summing up the DALY for each cycle and getting the average. [54]. The difference between the no intervention DALY and the DALY of each of the other interventions constituted the DALYs averted. [54].

The comparative efficiency of the strategies was measured using an incremental cost-effectiveness ratio (ICER). The ICER was calculated, defined as the difference in cost between the ‘no screening’ scenario and the intervention scenarios, divided by the change in DALY, this signified the average additional cost associated with 1 additional unit of DALY averted [54]. A willingness-to-pay threshold of 0.52 times GDP per capita, suggested by the University of York, was utilized in our analysis [57]. The willingness-to-pay threshold was estimated to be 0.52 times the GDP per capita of US$2065.75 (₦826,300) in Nigeria in 2021. In this case, an intervention with an ICER of less than US$1074.19/DALY (₦429676/DALY) is therefore an indication that mammography screening for Nigerian women aged 40–69 years, compared with no screening, is cost-effective.

To explore the effect of parameter uncertainty, we conducted univariate and probabilistic sensitivity analyses (PSA). The parameters included in the univariate sensitivity analysis were as follows: age-specific incidence, mammography sensitivity, mammography specificity, transition probabilities for the health states, stage-specific mortality rates, mammography screening cost, treatment cost, and discount rate. We used the lower and upper limits at a 95% confidence interval [54]. For parameters without confidence intervals, ± 30% of the base case was used as a conservative estimate. We varied each parameter individually to assess its impact on the overall results presented through a tornado diagram. In the PSA, all variables were varied simultaneously to further explore overall parameter uncertainty. Ten thousand iterations of Monte Carlo simulations were conducted, and a random value was drawn from each distribution, for each scenario, and the net health benefits calculated [54]. The distribution of each parameter was defined based on the literature (Table 1). We plotted the cost-effectiveness acceptability curves (CEAC) to show the proportion of simulations for which the intervention was cost-effective at different willingness-to-pay thresholds. In addition, the cost-effective acceptability frontier (CEAF) was used to explore the comparative efficiency of the interventions. This presented the possibility of any intervention being optimal when compared to all other competing choices [54]. Sixty-one repetitions of the simulations were conducted for different willingness-to-pay threshold ratios [54]. The possibility of any intervention being optimally the cost-effective compared to other competing interests, for each repetition, was evaluated for all the other interventions from the net monetary benefits (NMB) [54].

### Ethics consideration

An ethics statement was not required for this work.

## Results

The ‘no screening’ scenario had the least cost per patient (US$18.90 [₦7558]) when compared to the other interventions, resulting to a mortality rate of 623 per 100,000 simulated women. The annual screening had the highest cost (US$238.60 [₦95441]) and averted the highest DALY, with a mortality rate of 247 per 100,000 simulated women (Table 3).

**Table 3:** The cost, outcome, and incremental cost-effectiveness ratio of the interventions.

Comparators	Costs per case ($)	DALY lost	Incremental costs	Incremental DALY Averted	ICER ($/DALY averted)	Mortality rates (per 100,000)
No screening	18.896	1.899	–	–	–	623
Annual screening	238.603	0.839	219.707	1.060	207.241	247
Biennial screening	149.181	1.148	130.285	0.752	173.357	349

DALY – Disability adjusted life years.

ICER – Incremental cost-effectiveness ratio.

Table 3 showed that the ICER of annual screening and biennial screening interventions relative to the ‘no screening’ scenario was less than 0.52 times the GDP per capita [54] of Nigeria being $2065.75 in 2021. Hence, the annual and biennial screening interventions were all cost-effective. The annual screening intervention had the highest ICER below the WTP threshold and the highest NMB, thus the most efficient in maximizing resource utilization.

The scenario with a 50% participation rate was also considered cost-effective, with annual intervention having the highest ICER, same value as the 100% participation rate.

**Fig 2:**
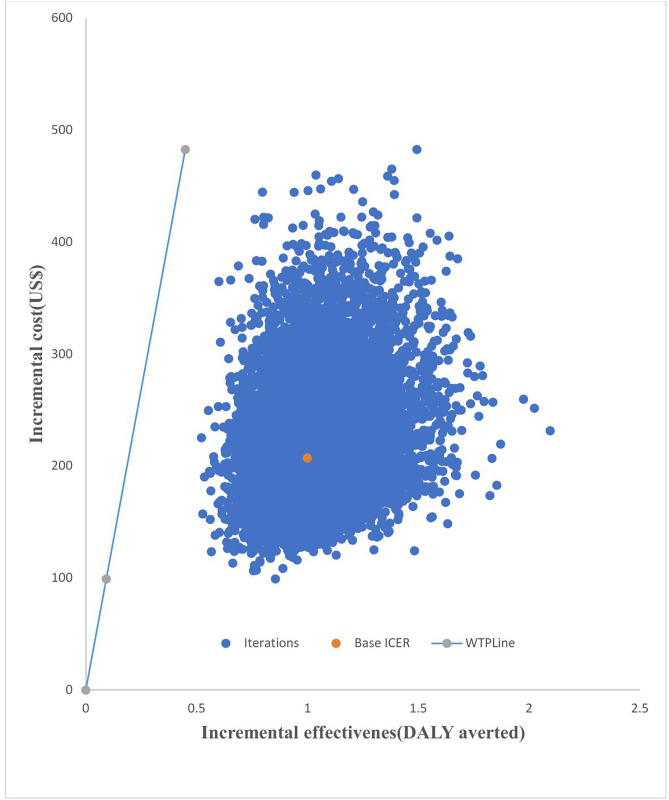
Cost-effectiveness plane of the annual screening intervention.

The PSA showed that the ICER result was not sensitive to the parameters, as all the simulations were below the cost-effectiveness threshold of US$1,074.19/DALY (₦429,676/DALY) averted. Fig 2 presents the scattered plot of the cost-effectiveness plane from the PSA, showing the uncertainty around the costs and outcomes. Annual mammography intervention was cost-effective, delivering results in the northeast quadrant, in which the intervention generates more health gains but is more expensive.

**Fig 3:**
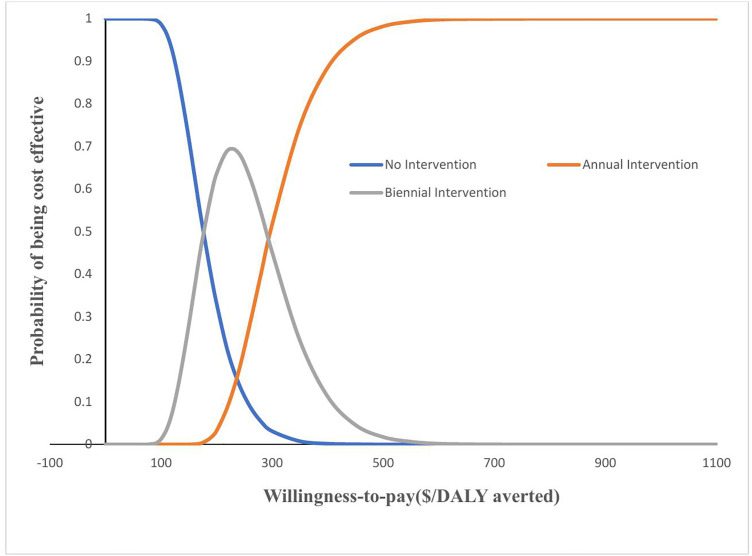
A cost-effectiveness acceptability curve showing the probability of the interventions being cost-effective at different willingness-to-pay thresholds.

Fig 3 presents the cost-effectiveness acceptability curve, a graph summarizing the impact of uncertainty on the results in relation to possible values of the cost-effectiveness threshold. In other words, it shows the probability of the intervention being cost-effective at different willingness-to-pay thresholds.

**Fig 4:**
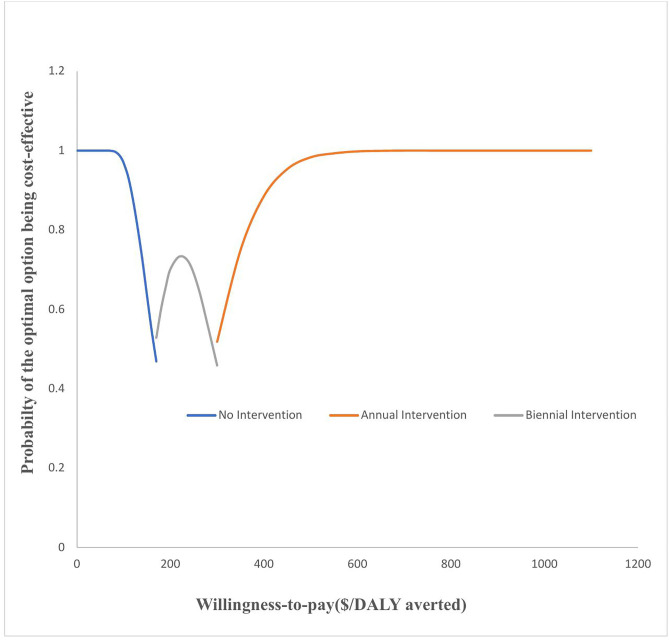
A cost-effectiveness acceptability frontier showing the decision uncertainty surrounding the optimal choice.

Fig 4 illustrates the relative efficiency of the interventions in optimizing healthcare resource allocation. Under parameter uncertainty and willingness-to-pay values, the CEAF showed which intervention had the highest probability of being cost-effective. Specifically, it shows the decision uncertainty surrounding the optimal choice. The ‘no intervention’ scenario showed the highest probability of being cost-effective if the payer is not willing to pay any amount. With a willingness-to-pay value of at least ₦68,000 ($170) to avert a DALY, the biennial intervention had the highest probability of being cost-effective. On the other hand, with a willingness-to-pay value of more than ₦120,000 ($300) to avert one extra DALY, the annual intervention had the highest probability of being cost-effective.

**Fig 5:**
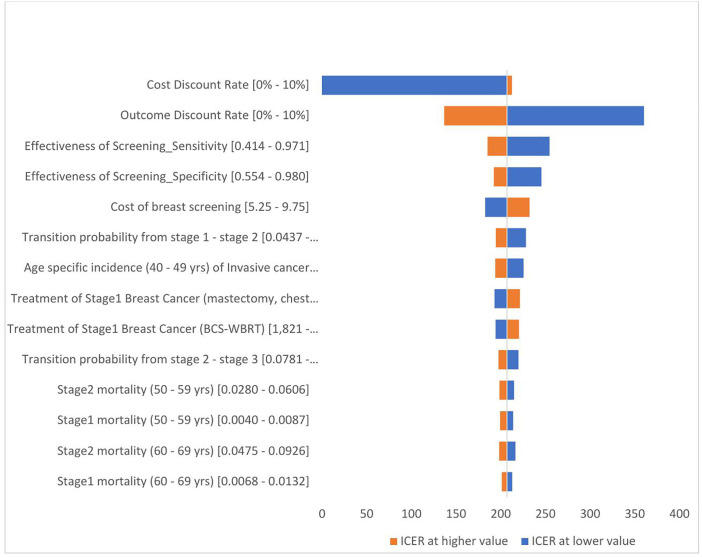
A tornado diagram showing the uncertainty impact of key parameters on the ICER of the most cost-effective approach.

The univariate sensitivity analysis showed that the cost discount rate was the most influential parameter, followed by the outcome discount rate. At the lower limit of discount rate (0), the ICER reduced from ₦82,896/DALY ($207.24/DALY) averted to ₦0/DALY ($0/DALY), whereas at the higher limit (0.1), the ICER increased to $212.42/DALY. Breast screening cost and treatment costs for stage 1 breast cancer had an insignificant effect on the result. Details are shown in Fig 5.

## Discussion

This study is the first to provide insight into the cost-effectiveness of mammography screening for Nigerian women. Our findings provide initial comparative evidence on the cost-effectiveness of alternative mammography screening scenarios for Nigerian women from a healthcare payer’s perspective. Within the assumptions of the model, several screening strategies were estimated to be cost-effective relative to no screening. Although annual screening emerged as the most favorable option in the model, this finding is contingent on model structure and input assumptions and should not be interpreted as a definitive recommendation regarding screening intervals. Further empirical evidence is needed to inform national screening policy.

Although our ICER value (US$207.24 per DALY averted) was lower than those reported in studies from some developing countries, our findings were consistent in demonstrating that mammography is a cost-effective intervention. Nguyen and Adang (2018) found mammography screening to be cost-effective for women aged 50–59 years, with ICERs of US$3,647.06 per life year gained for women aged 50–54 years, and US$4,405.44 per life year gained for women aged 55–59 years, based on a threshold of three times the Vietnamese GDP per capita [44]. A study by Zehtab *et* al., (2016) found mammography screening to be a cost-effective intervention among Iranian women aged 35–69 years with an ICER of US$6,264 per DALY averted [15]. Contrary to our findings, Zelle et al. (2012) reported that mammography screening was not a cost-effective intervention for Ghanaian women aged 40–69 years, with a cost of US$12,908 per DALY averted [16]. This could be a result of both patient-level and program-level costs incorporated in the study. Unlike our study, which used only patient-level costs and a macro-costing technique, the other study employed an ingredient-based approach to cost analysis. This more granular method itemizes each resource used, which can result in higher overall cost estimates compared to aggregated costing methods. Also, exploring an alternative scenario of 50% participation rate considered mammography screening to be cost-effective with annual interval being the most optimal intervention. This could be due to the non-consideration of actual implementation costs for the interventions.

This study has implications for national health policy, as the findings suggest that mammography screening should be considered for inclusion in the national screening program, in line with the NSCCP goal. Such an initiative could optimize healthcare expenditure, as the preventative screening and early intervention will result in a reduced need for expensive treatments. Currently, there is no national mammography screening. Some Insurers, like the NHIA and Health Maintenance Organizations (HMOs), include mammograms in their healthcare packages; however, this service is not accessible to all eligible women, and overall health insurance coverage in Nigeria is still low, at about 10% [40]. While it is encouraging for HMOs and other private insurers to consider the inclusion of mammography screening in their comprehensive healthcare packages, a national mammography program would likely reduce inequalities in screening access and participation. Further studies, such as budget impact analyses, are needed to inform policymakers and healthcare payers about the financial feasibility of adopting mammography screening within budgetary realities.

The potential to reduce breast cancer mortality depends on the frequency of mammography screening rates and facilitating early-stage detection. Based on our findings, the implementation of mammography screening for Nigerian women aged 40–69 years warrants consideration, provided that key system-level conditions are met. With the modeled age group and screening interval deviating from the International Agency for Research on Cancer (IARC) recommendations, there would be a need for policy makers to deliberate on aligning with or justifying deviations from the guidance. Effective implementation would require a sufficiently resourced health system with sustainable financing to support screening, timely diagnosis, and appropriate treatment, including adequate equipment, infrastructure, trained workforce, quality assurance mechanisms, and ongoing monitoring processes. A clearly defined administrative structure would be necessary to ensure accountability for program implementation, quality assurance, and evaluation. In addition, validated protocols should govern all stages of the screening pathway, including identification of the target population, invitation and participation of eligible women, performance and quality control of screening tests, referral mechanisms, and subsequent diagnostic and treatment services. Successful implementation would also depend on culturally appropriate communication and education for both women and healthcare providers, delivering balanced and objective information on the potential benefits and harms of mammography screening. Adherence to established guidelines for screening, diagnosis, and treatment, supported by a robust information system capable of capturing data across the entire screening continuum, would be essential. Finally, regular monitoring, evaluation, and transparent reporting of program performance and impact should be conducted using outcome indicators that include both clinical outcomes and women’s safety and satisfaction.

Before national-scale implementation, state-level implementation pilot trials should be conducted to assess key outcomes, including acceptability, adoption, appropriateness, costs, feasibility, fidelity, penetration, and sustainability [58]. Invitation centers would be tasked with inviting and scheduling eligible women for participation, while the screening service would encompass the full diagnostic continuum [59]. To enhance awareness and participation, client-oriented interventions, such as mass media campaigns, small media, group education, and the engagement of community health workers, should be deployed. This is critical, as low participation rates diminish the cost-effectiveness of screening programs [15]. While exploring sustainable financing mechanisms to support mammography screening, it is urgent for the government to prioritize healthcare financing by increasing total healthcare expenditure and integrating cancer screening and management into comprehensive health insurance packages. Such measures would reduce the costs and out-of-pocket payments associated with breast cancer screening and management, which remain significant barriers to women’s participation in screening programs.

Limitations of the study include the paucity of data on mammography sensitivity for age-specific groups of Nigerian women; hence, we used data for the population. We applied transition probabilities, the relative risk of invasive cancer in DCIS, and health utilities from other countries, and assumed they applied to Nigeria. However, we explored the uncertainty in the sensitivity analysis. In addition, we did not include the non-medical costs and implementation costs, such as the cost of identifying eligible women, administrative costs, and other ancillary costs, due to a lack of realistic country-specific or related data. We also tried to avoid overestimation of cost due to unrealistic cost estimates. Furthermore, we only explored mammography screening at the start age of 40 years. While IARC guidelines recommend screening initiation at age 50, emerging regional evidence and national burden data have informed context-adapted approaches in low- and middle-income settings. There are increasing evidence of relative reduction in breast cancer mortality with commencement of mammography screening at this age [60–63], and early breast cancer cases in Africa; a meta-analysis showed that 58% of diagnosed patients were less than 50 years [64]. Also, Nigeria’s relatively young median age (18–19 years) and the substantial proportion of the adult population in the 40–49 age bracket provided a demographic rationale for modeling this age group [65], though it falls outside IARC’s standard recommendation. The model assumes prompt initiation and completion of appropriate treatment following diagnosis. This represents a best-case scenario and does not reflect real-world variability in access to care, treatment delays, and incomplete treatment in Nigeria. As a result, the model may overestimate the health benefits of screening. Future modelling work should incorporate stage-specific treatment coverage, delays, and incomplete treatment pathways as data become available, and that our findings should be interpreted in this context. Furthermore, the model description did not specify how adherence across rounds, diagnostic follow-up, completion after abnormal screens, and loss-to-follow-up are represented in the base case due to data limitation.

## Conclusion

This modelling-based cost-effectiveness analysis suggests that mammography screening strategies may be cost-effective from the healthcare payer’s perspective under the assumptions of the model. Differences in cost-effectiveness were observed across screening intervals, highlighting the need for further evidence, including budget impact and feasibility analyses, to inform screening policy decisions in Nigeria.
