# Supplementary material for: Cost-effectiveness analysis of mammography screening for early detection of breast cancer in Nigeria
Source: PLoS One. 2026 Jun 12;21(6):e0351492. doi: 10.1371/journal.pone.0351492 (PMC13262826; doi:10.1371/journal.pone.0351492)
Supplement: Attachment — Submitted filename: Response to Reviewers.docx [file pone.0351492.s002.docx]

**Editor**

1. Please ensure that your manuscript meets PLOS ONE's style requirements, including those for file naming. The PLOS ONE style templates can be found at https://journals.plos.org/plosone/s/file?id=wjVg/PLOSOne_formatting_sample_main_body.pdf and https://journals.plos.org/plosone/s/file?id=ba62/PLOSOne_formatting_sample_title_authors_affiliations.pdf.

Thank you for taking your time to review our manuscript.

We have formatted the manuscript to meet PLOS One’s style requirement, particularly those for file naming.

2. Please note that PLOS One has specific guidelines on code sharing for submissions in which author-generated code underpins the findings in the manuscript. In these cases, we expect all author-generated code to be made available without restrictions upon publication of the work. Please review our guidelines at <https://journals.plos.org/plosone/s/materials-and-software-sharing#loc-sharing-code> and ensure that your code is shared in a way that follows best practice and facilitates reproducibility and reuse.

We have deposited our data in Mendeley Data at <https://data.mendeley.com/datasets/5k9zwnz4yb/1>.

3. We noticed you have some minor occurrence of overlapping text with the following previous publication(s), which needs to be addressed:

<https://bmjpublichealth.bmj.com/content/2/2/e001356>.

<https://journals.plos.org/plosntds/article?id=10.1371%2Fjournal.pntd.0006124>

In your revision ensure you cite all your sources (including your own works), and quote or rephrase any duplicated text outside the methods section. Further consideration is dependent on these concerns being addressed.

We have addressed the occurrence of overlapping texts and cited all our sources, especially my works.

4. Thank you for stating in your Funding Statement:

“This study was partly funded by the Royal Society of Tropical Medicine and Hygiene (RSTMH) in partnership with the National Institute for Health Research (NIHR), 2021 Small Grants Programme. The funders had no role in study design, data collection and analysis, decision to publish, or preparation of the manuscript.”

Please provide an amended statement that declares *all* the funding or sources of support (whether external or internal to your organization) received during this study, as detailed online in our guide for authors at <http://journals.plos.org/plosone/s/submit-now>.  Please also include the statement “There was no additional external funding received for this study.” in your updated Funding Statement.

Please include your amended Funding Statement within your cover letter. We will change the online submission form on your behalf.

The funding statement was amended as follows, “This study was mostly funded by the Royal Society of Tropical Medicine and Hygiene (RSTMH) in partnership with the National Institute for Health Research (NIHR), 2021 Small Grants Programme. The corresponding author, IJN, funded some parts of the study. The external funders had no role in study design, data collection and analysis, decision to publish, or preparation of the manuscript.”

5. Please provide a complete Data Availability Statement in the submission form, ensuring you include all necessary access information or a reason for why you are unable to make your data freely accessible. If your research concerns only data provided within your submission, please write "All data are in the manuscript and/or supporting information files" as your Data Availability Statement.

The data availability statement was re-written as follows, “All data and code are available in the Mendeley Data at <https://data.mendeley.com/datasets/5k9zwnz4yb/1>(DOI: 10.17632/5k9zwnz4yb.1).”

6. Please be informed that funding information should not appear in the Acknowledgments section or other areas of your manuscript. We will only publish funding information present in the Funding Statement section of the online submission form. Please remove any funding-related text from the manuscript.

The funding statement has been removed from the manuscript.

7. If the reviewer comments include a recommendation to cite specific previously published works, please review and evaluate these publications to determine whether they are relevant and should be cited. There is no requirement to cite these works unless the editor has indicated otherwise.

Not applicable

**Reviewer 1**

1. The Markov model you’ve used is appropriate, but several of the input values—like transition probabilities and utility scores—are taken from studies outside Nigeria or are outdated. It would help to either source more locally relevant data or explain clearly why these were used.

Thank you for taking your time to review our manuscript.

We used country-specific data were available and used data for countries with similar health characteristics when not available. This was acknowledged in the study limitation. However, we explored the uncertainties in the data utilized using the sensitivity analysis.

2. Assuming 100 percent screening participation is unrealistic in the Nigerian context. Please try modeling lower compliance rates or at least discuss how this assumption affects the results.

The reviewer is right. As 100 percent screening participation is unrealistic in Nigeria, we have now modeled a 50 percent participation rate and updated the manuscript accordingly.

3. The sensitivity analysis section feels a bit thin. You could improve this by explaining the range used for each variable and maybe adding a tornado diagram to show which factors influence the outcome most.

Thank you for this observation. The sensitivity analysis section includes a tornado diagram that captured the ranges used for the various variables. However, they were not included in the manuscript as stipulated by the journal guidelines, rather it was updated as a separate file. Also, the distribution of the variables was already discussed under the analysis section of the methodology and shown in Table 1.

4. There's no mention of ethical approval or waiver. Even though it’s a modeling study, this should be clarified in the manuscript.

We followed the journal guidelines on the submission for a study not involving human or animal subjects. Hence, we have included that “an ethics statement was not required for this work” in the method section.

5. The underlying data and model files haven’t been made publicly available. Please consider sharing them in a repository so others can replicate or build on your work.

We have deposited our data set in the Mendeley Data and updated the manuscript accordingly.

6. The discussion would benefit from addressing real-world implementation challenges -things like infrastructure, funding, or public awareness that affect whether screening programs can actually be rolled out.

Thank you for this comment. We have included solutions to real-world implementation challenges that could emerge (see page 22).

**Reviewer 2**

1. If the primary objective of the manuscript is a cost-effectiveness analysis, it remains unclear why the authors extend their conclusions to the definition of screening guidelines, particularly with respect to starting age and screening interval.
   We have now revised the aim and conclusion for clarity. The study aimed to assess the cost-effective and optimal screening interval of mammography screening.
2. The statement “Currently, there is no national breast cancer mammography screening program in Nigeria; hence, no national screening guidelines” (lines 58–59) is conceptually problematic. Guidelines do not necessarily follow the existence of a screening program. They may either be developed based on robust evidence tailored to a specific national context or be adapted from existing international guidance. In this manuscript, the authors themselves cite international recommendations (e.g. IARC) that, at a global level, recognize biennial mammography starting at age 50 for women at average risk (references 62–63). The rationale for departing from these established recommendations is not sufficiently justified.
   This reviewer is right, and we have now revised our statement in line with the reviewer’s suggestion. We acknowledge that although there is currently no screening program in the country, clinicians depend on international guidelines to screen their patients, which may be the best option for the patients. Therefore, we have now deleted the claim that there is “no screening guideline”.
3. The authors state that evaluating the cost-effectiveness of a national mammography screening program is crucial to ensure accessibility and that a modelling approach is appropriate given the lack of feasibility of trial-based evaluations in Nigeria. While this argument supports the use of modelling, it does not adequately explain how the authors arrive at a recommendation of annual mammography starting at age 40, which is not aligned with IARC recommendations. A cost-effectiveness model alone cannot replace the evidentiary basis required to define screening age and interval.
   We thank the reviewer for this important clarification and agree that our original wording overstated the implications of the cost-effectiveness analysis. Randomised controlled trial-based economic evaluations remains the gold standard for establishing screening age and interval, and a modelling study alone cannot substitute for the evidentiary foundation required to define national screening policy. We also acknowledge that our cost-effectiveness analysis does not, in itself, ensure accessibility as previously claimed, but rather provides supportive economic evidence to inform decision making alongside considerations of feasibility, equity, and health system capacity.

In our model, age 40 and annual screening was included as pre-specified exploratory scenarios to allow comparison across plausible screening strategies, rather than to recommend an optimal national policy. Although the model identified annual screening from age 40 as cost-effective under certain assumptions, this finding does not constitute Level 1 evidence and should not be interpreted as a policy recommendation, particularly given its divergence from IARC guidance.

We have now revised the manuscript to clarify that the purpose of the modelling analysis is to assess the value for money of alternative screening strategies, not to define the appropriate starting age or screening interval. The revised text now reads:

*“…it is crucial to evaluate the value for money of a national mammography screening in Nigeria to support decision making. While a randomised controlled trial-based economic evaluation would be ideal, such studies may not currently be feasible. In this context, a robust modelling approach can provide a feasible complementary source of evidence to compare alternative screening scenarios.”*

4. In addition, the model relies on DALYs as the primary outcome. It is generally accepted that cancer screening programs should primarily be evaluated on mortality reduction, as earlier diagnosis without a demonstrated effect on mortality is often considered a key argument against screening adoption. The use of DALYs may therefore limit the ability of the model to inform screening policy decisions, particularly with regard to overdiagnosis and overtreatment.
   We agree on the importance of mortality reduction as an outcome for cancer screening. We have now provided this evidence as a secondary outcome. No intervention = 6,447 deaths (623 deaths per 100,000 population); Annual = 2,557 death (247 deaths per 100,000 population); Biennial = 3,616 deaths (349 deaths per 100,000 population). This has been included in the manuscript.
5. A further concern relates to the assumption (line 129) that all breast cancer patients undergo prompt and adequate treatment. This assumption appears inconsistent with the authors’ own introductory statement (line 55) highlighting that early diagnosis of breast cancer in Nigeria faces multiple challenges, resulting in late presentation. The implementation of a screening program presupposes the availability of timely and effective diagnostic and treatment pathways, which are not clearly demonstrated in the manuscript.
   The model presumed that all breast cancer patients underwent prompt and adequate treatment. This was supposedly so, because the model assumed an effective screening approach in place, which will ensure prompt and adequate screening. Given that this may not be absolutely feasible in a setting like Nigeria, we have discussed this a limitation in our model in the Discussion section.
6. It is therefore difficult to understand how the cost-effectiveness model can robustly support conclusions such as those presented in lines 334 and 341, where mammography screening is described as cost-effective from the healthcare payer’s perspective and annual screening is identified as the optimal interval. These conclusions appear to go beyond what can reasonably be inferred from the modelling framework.
   Thank you for this important comment. We agree that the original wording overstated the strength of the conclusions that can be drawn from the modelling framework. The cost-effectiveness model was designed to **compare alternative screening scenarios under a defined set of assumptions,** rather than to establish definitive screening intervals or policy recommendations. We have therefore revised the relevant sections to clarify that, while mammography screening scenarios were cost-effective from the healthcare payer’s perspective within the model, the identification of annual screening as the most favorable option reflects **model-specific results** rather than a robust or prescriptive conclusion. These findings should be interpreted as exploratory and hypothesis-generating. The revised text (page 20) now emphasizes that the analysis provides **comparative economic insights** to inform future research and policy discussions, rather than definitive guidance on screening intervals.
7. The authors also state that “the potential to reduce breast cancer mortality depends on increasing mammography screening rates and facilitating early-stage detection” (line 366). This assertion remains debated in the literature (see, for example, Autier et al., Evaluation of screening mammography effectiveness: The IARC recommendations of 2015 need revision, European Journal of Cancer, 2025). In this context, it is difficult to justify recommendations favoring mammography screening for Nigerian women aged 40–69 based on a DALY-based cost-effectiveness analysis that is not consistent with IARC guidance.
   We agree with the reviewer. We have now revised the statement as shown below:

*“The potential to reduce breast cancer mortality depends on the frequency of mammography screening rates and facilitating early-stage detection”.*

8. Finally, the statement regarding “current evidence on the increasing incidence of breast cancer in women aged 40–49” (lines 389–391) requires clearer justification and stronger referencing. At present, much of the observed increase in incidence in this age group is attributable to the introduction of screening itself, particularly in settings where screening was previously absent or has been recently extended to younger women. This phenomenon does not provide evidence of screening effectiveness and is widely regarded as a manifestation of overdiagnosis, with consequent overtreatment.

We agree that Increase in incidence in this age group is due to the introduction of screening itself. We intended to write on the relative reduction in breast cancer mortality with the start of screening in this age group. With strong references, we have now revised the statement as follows: “This is due to the increasing evidence of relative reduction in breast cancer mortality with commencement of mammography screening at this age”

**Reviewer 3**

The manuscript evaluates the cost-effectiveness of annual versus biennial mammography for Nigerian women starting at age 40 using a lifetime Markov model with DALYs and 5% discounting. The question is policy-relevant and aligned with Nigeria’s cancer-control priorities, and the overall analytic approach is reasonable for screening evaluation. The authors also make a good-faith attempt to characterize uncertainty through sensitivity analyses and acceptability outputs, and they draw on Nigerian inputs where available, which improves local relevance.

However, several issues need to be corrected or more rigorously handled before the findings can be interpreted confidently for policy. First, the stated mortality structure—namely that only stage IV patients can die from breast cancer—is not clinically plausible and is likely to distort life-years/DALYs and therefore the ICER; moreover, the manuscript appears internally inconsistent on this point given the inclusion of stage-specific fatality parameters, so the authors should clarify what was actually implemented. In either case, the model should be revised to a realistic stage-specific mortality framework (or calibrated to observed survival), and the impact of alternative mortality structures should be explored in scenario analyses.

Thank you for your comments.

Regarding the mortality structure, the model applied what the reviewer proposed. The error was from the diagram arrow, which originates from Stage IV alone. The outer rectangular box in the diagram was meant to demonstrate that death from cancer or natural death can occur from any stage. We have now edited the arrow to emanate from the outer rectangular box.

Second, the model presumes that all breast cancer patients receive prompt and adequate treatment, which does not reflect real-world variability in access, timeliness, and completion of care in Nigeria and may overstate screening benefits. The analysis should incorporate scenarios with constrained access, treatment delays, and incomplete treatment (including stage-specific treatment coverage if possible), and clearly report how these change outcomes and conclusions.

We thank the reviewer for highlighting this important limitation. We agree that assuming prompt and complete treatment following diagnosis does not fully reflect real-world variability in access, timeliness, and completion of breast cancer care in Nigeria and may overestimate the benefits of screening in practice.

Due to the absence of reliable, stage-specific national data on treatment initiation, delays, and completion rates, we were unable to parameterize constrained-access scenarios in the current model without introducing additional unverified assumptions. We have therefore retained the simplified treatment assumption to preserve internal consistency of the model, while explicitly acknowledging its implications.

We have revised the Discussion to clarify that the estimated cost-effectiveness of screening reflects a best-case treatment scenario and that reduced treatment access or delays would likely attenuate health gains and worsen cost-effectiveness. We now emphasize that future modelling work should incorporate stage-specific treatment coverage, delays, and incomplete treatment pathways as data become available, and that our findings should be interpreted in this context.

Third, the model description should explicitly specify how screening uptake, adherence across rounds, diagnostic follow-up completion after abnormal screens, and loss-to-follow-up are represented in the base case. If any of these are not modeled or are implicitly assumed to be perfect (e.g., 100% uptake/adherence/follow-up), that should be stated plainly and tested with plausible ranges because these parameters strongly influence both effectiveness and cost-effectiveness—particularly in settings where participation and follow-up can be substantial bottlenecks.

The model has now explored an alternative scenario of 50% screening uptake. However, the model description did not specify how adherence across rounds, diagnostic follow-up completion after abnormal screens, and loss-to-follow-up are represented in the base case due to data limitation. To avoid uncertain data, address this concern, we have discussed this as a limitation in the discussion section.

Fourth, the exclusion of implementation and other non-medical program costs (e.g., outreach, invitation/recall systems, administration, quality assurance, patient navigation, and program management) will tend to bias results in favor of screening. Even if Nigeria-specific estimates are limited, the authors should include plausible ranges informed by comparable programs and conduct scenario analyses to assess whether conclusions remain robust once these costs are incorporated. Finally, the current data/model availability statement is unlikely to enable full replication of a lifetime decision model. Sharing the model file/code and providing a complete parameter table (including all transition probabilities, screening and follow-up pathways, treatment assumptions, the full costing build-up, and sources) as supplementary material would substantially strengthen transparency, reproducibility, and confidence in the results.

We agree with the concerns raised about the exclusion of implementation and other non-medical program costs. Lack of realistic data made it impossible to include these costs. We also try to avoid overestimation of cost due to unrealistic cost estimates. Hence, we have discussed this limitation in the discussion section.

**Reviewer 4**

1. It would be appropriate to highlight more clearly the originality of the study by emphasizing how it differs from and adds to existing studies.

Thank for taking your time to review our manuscript. Currently, there is no published Nigerian study on the subject. Hence, this study is the first to provide insights into the cost effectiveness of mammography screening for Nigerian women. This was stated in the manuscript.

2. In addition, it would be appropriate to describe more clearly—also through a dedicated paragraph—the characteristics of the Nigerian healthcare system and of mammography screening.

Thank you for your suggestion. A dedicated paragraph for the characteristics of the Nigerian healthcare system and mammography screening has been included.

3. Finally, it would also be appropriate to highlight potential future directions for the research.

Thank you for this comment. We stated that “Further studies, such as budget impact analyses, are needed to inform policymakers and healthcare payers about the financial feasibility of adopting mammography screening within budgetary realities.”
