# Supplementary material for: Cost-effectiveness analysis of mammography screening for early detection of breast cancer in Nigeria
Source: PLoS One. 2026 Jun 12;21(6):e0351492. doi: 10.1371/journal.pone.0351492 (PMC13262826; doi:10.1371/journal.pone.0351492)
Supplement: Attachment — Submitted filename: Response_to_Reviewers_auresp_2.docx [file pone.0351492.s003.docx]

**Journal Requirements:**

1. If the reviewer comments include a recommendation to cite specific previously published works, please review and evaluate these publications to determine whether they are relevant and should be cited. There is no requirement to cite these works unless the editor has indicated otherwise.

Not applicable.

2. Please review your reference list to ensure that it is complete and correct. If you have cited papers that have been retracted, please include the rationale for doing so in the manuscript text, or remove these references and replace them with relevant current references. Any changes to the reference list should be mentioned in the rebuttal letter that accompanies your revised manuscript. If you need to cite a retracted article, indicate the article’s retracted status in the References list and also include a citation and full reference for the retraction notice.

Thank you for taking your time to review our manuscript.

We have reviewed our reference list for completeness and correctness. We included additional references (64 and 65) to support our point on target age for the model.

**Reviewer #2:**

3. Overall, the revisions introduced by the authors respond satisfactorily to the previous comments and represent a meaningful improvement of the manuscript. It is understandable that the modelling approach focuses on the 40–49 age group, which currently does not comply with international recommendations that generally indicate the start of screening at age 50. This limitation is correctly acknowledged in the manuscript but the issue might be further contextualized not only by recalling references 60–63, which, in my opinion, do not have the authority to be considered a valid alternative to IARC recommendations, but also by considering perhaps the demographic characteristics of Nigeria, whose relatively young median age may partly support the authors’ focus on the 40–49 age group. Nevertheless, the question of the transferability of a purely economic modelling analysis to a real-world implementation context remains open, particularly when the target age group and screening frequency differ from those universally recommended by IARC. In this respect, the practical implementation considerations discussed on page 22 add value to the manuscript. On the one hand, they outline possible pathways for translating the theoretical modelling results into real-world practice; on the other hand, they appropriately acknowledge the limitations inherent to a theoretical economic model when informing screening policy decisions.

Thank you once more for reviewing our manuscript. We deeply appreciate your suggestion about Nigeria’s demographics.

We never intended to present references 60-63 as equivalent alternatives to IARC’s guidance as it remains an international consensus. The references are evidence supporting context-specific flexibility. We have now revised the manuscript (page 24) and it reads:

“*While the International Agency for Research on Cancer (IARC) guidelines recommend screening initiation at age 50, emerging regional evidence and national burden data have informed context-adapted approaches in low- and middle-income settings. There are increasing evidence of relative reduction in breast cancer mortality with commencement of mammography screening at this age [60–63], and early breast cancer cases in Africa; a meta-analysis showed that 58% of diagnosed patients were less than 50 years [64]. Also, Nigeria’s relatively young median age (18-19 years) and the substantial proportion of the adult population in the 40-49 age bracket provided a demographic rationale for modeling this age group [65], though it falls outside IARC’s standard recommendation.*”

Thank you for appreciating the value of the practical implementation considerations. Regarding the age group and screening frequency deviating from IARC guidance, we have included the following in the manuscript (page 22):

*“With the modeled age group and screening interval deviating from the International Agency for Research on Cancer (IARC) recommendations, there would be a need for policy makers to deliberate on aligning with or justifying deviations from the guidance.*”
