# Supplementary material for: Cost-effectiveness analysis of mammography screening for early detection of breast cancer in Nigeria
Source: PLoS One. 2026 Jun 12;21(6):e0351492. doi: 10.1371/journal.pone.0351492 (PMC13262826; doi:10.1371/journal.pone.0351492)
Supplement: Peer review history

# Peer review: Decision Letter 0

**DOI:** 10.1371/journal.pone.0351492.r001

**Authors:** Daniele Ugo Tari

2 Jan 2026

PONE-D-25-51694

Cost-effectiveness analysis of mammography screening for early detection of breast cancer in Nigeria

PLOS One

Dear Dr. Nduka,

Thank you for submitting your manuscript to PLOS ONE. After careful consideration, we feel that it has merit but does not fully meet PLOS ONE’s publication criteria as it currently stands. Therefore, we invite you to submit a revised version of the manuscript that addresses the points raised during the review process.

Please submit your revised manuscript by Feb 16 2026 11:59PM. If you will need more time than this to complete your revisions, please reply to this message or contact the journal office at plosone@plos.org. When you're ready to submit your revision, log on to https://www.editorialmanager.com/pone/ and select the 'Submissions Needing Revision' folder to locate your manuscript file.

Please include the following items when submitting your revised manuscript:

- A letter that responds to each point raised by the academic editor and reviewer(s). You should upload this letter as a separate file labeled 'Response to Reviewers'.
- A marked-up copy of your manuscript that highlights changes made to the original version. You should upload this as a separate file labeled 'Revised Manuscript with Track Changes'.
- An unmarked version of your revised paper without tracked changes. You should upload this as a separate file labeled 'Manuscript'.

If you would like to make changes to your financial disclosure, please include your updated statement in your cover letter. Guidelines for resubmitting your figure files are available below the reviewer comments at the end of this letter.

If applicable, we recommend that you deposit your laboratory protocols in protocols.io to enhance the reproducibility of your results. Protocols.io assigns your protocol its own identifier (DOI) so that it can be cited independently in the future. For instructions see: https://journals.plos.org/plosone/s/submission-guidelines#loc-laboratory-protocols. Additionally, PLOS ONE offers an option for publishing peer-reviewed Lab Protocol articles, which describe protocols hosted on protocols.io. Read more information on sharing protocols at https://plos.org/protocols?utm_medium=editorial-email&utm_source=authorletters&utm_campaign=protocols.

We look forward to receiving your revised manuscript.

Kind regards,

Daniele Ugo Tari, M.D.

Academic Editor

PLOS One

Journal Requirements:

When submitting your revision, we need you to address these additional requirements.

1. Please ensure that your manuscript meets PLOS ONE's style requirements, including those for file naming. The PLOS ONE style templates can be found at

https://journals.plos.org/plosone/s/file?id=wjVg/PLOSOne_formatting_sample_main_body.pdf and

https://journals.plos.org/plosone/s/file?id=ba62/PLOSOne_formatting_sample_title_authors_affiliations.pdf.

2. Please note that PLOS One has specific guidelines on code sharing for submissions in which author-generated code underpins the findings in the manuscript. In these cases, we expect all author-generated code to be made available without restrictions upon publication of the work. Please review our guidelines at https://journals.plos.org/plosone/s/materials-and-software-sharing#loc-sharing-code and ensure that your code is shared in a way that follows best practice and facilitates reproducibility and reuse.

3. We noticed you have some minor occurrence of overlapping text with the following previous publication(s), which needs to be addressed:

https://bmjpublichealth.bmj.com/content/2/2/e001356.

https://journals.plos.org/plosntds/article?id=10.1371%2Fjournal.pntd.0006124

In your revision ensure you cite all your sources (including your own works), and quote or rephrase any duplicated text outside the methods section. Further consideration is dependent on these concerns being addressed.

4. Thank you for stating in your Funding Statement:

“This study was partly funded by the Royal Society of Tropical Medicine and Hygiene (RSTMH) in partnership with the National Institute for Health Research (NIHR), 2021 Small Grants Programme. The funders had no role in study design, data collection and analysis, decision to publish, or preparation of the manuscript.”

Please provide an amended statement that declares *all* the funding or sources of support (whether external or internal to your organization) received during this study, as detailed online in our guide for authors at http://journals.plos.org/plosone/s/submit-now. Please also include the statement “There was no additional external funding received for this study.” in your updated Funding Statement.

Please include your amended Funding Statement within your cover letter. We will change the online submission form on your behalf.

5. Please provide a complete Data Availability Statement in the submission form, ensuring you include all necessary access information or a reason for why you are unable to make your data freely accessible. If your research concerns only data provided within your submission, please write "All data are in the manuscript and/or supporting information files" as your Data Availability Statement.

6. Please be informed that funding information should not appear in the Acknowledgments section or other areas of your manuscript. We will only publish funding information present in the Funding Statement section of the online submission form. Please remove any funding-related text from the manuscript.

7. If the reviewer comments include a recommendation to cite specific previously published works, please review and evaluate these publications to determine whether they are relevant and should be cited. There is no requirement to cite these works unless the editor has indicated otherwise.

Additional Editor Comments (if provided):

Dear Authors,

The manuscript addresses a policy-relevant question for Nigeria by modeling the cost-effectiveness of annual versus biennial mammography from age 40 using a lifetime Markov framework with DALYs and discounting, and it appropriately reports uncertainty analyses while drawing on local data where available. However, the authors need to address several issues before the findings can be interpreted confidently for policy. To be suitable for publication, the paper needs extensive revision.

I suggest to address all the reviewers' comments and to resubmit it.

Sincerely,

[Note: HTML markup is below. Please do not edit.]

Reviewers' comments:

Reviewer's Responses to Questions

**Comments to the Author**

1. Is the manuscript technically sound, and do the data support the conclusions?

The manuscript must describe a technically sound piece of scientific research with data that supports the conclusions. Experiments must have been conducted rigorously, with appropriate controls, replication, and sample sizes. The conclusions must be drawn appropriately based on the data presented.

Reviewer #1: Partly

Reviewer #2: No

Reviewer #3: Partly

Reviewer #4: Yes

**********

2. Has the statistical analysis been performed appropriately and rigorously?

Reviewer #1: Yes

Reviewer #2: I Don't Know

Reviewer #3: Yes

Reviewer #4: Yes

**********

3. Have the authors made all data underlying the findings in their manuscript fully available?

The PLOS Data policy requires authors to make all data underlying the findings described in their manuscript fully available without restriction, with rare exception (please refer to the Data Availability Statement in the manuscript PDF file). The data should be provided as part of the manuscript or its supporting information, or deposited to a public repository. For example, in addition to summary statistics, the data points behind means, medians and variance measures should be available. If there are restrictions on publicly sharing data—e.g. participant privacy or use of data from a third party—those must be specified.

Reviewer #1: No

Reviewer #2: Yes

Reviewer #3: No

Reviewer #4: Yes

**********

4. Is the manuscript presented in an intelligible fashion and written in standard English?

PLOS ONE does not copyedit accepted manuscripts, so the language in submitted articles must be clear, correct, and unambiguous. Any typographical or grammatical errors should be corrected at revision, so please note any specific errors here.

Reviewer #1: Yes

Reviewer #2: Yes

Reviewer #3: Yes

Reviewer #4: Yes

**********

5. Review Comments to the Author

Please use the space provided to explain your answers to the questions above. You may also include additional comments for the author, including concerns about dual publication, research ethics, or publication ethics. (Please upload your review as an attachment if it exceeds 20,000 characters)

Reviewer #1: Thank you for taking on a very relevant topic and using a modeling approach to explore the cost-effectiveness of breast cancer screening in Nigeria.

The Markov model you’ve used is appropriate, but several of the input values—like transition probabilities and utility scores—are taken from studies outside Nigeria or are outdated. It would help to either source more locally relevant data or explain clearly why these were used.

Assuming 100 percent screening participation is unrealistic in the Nigerian context. Please try modeling lower compliance rates or at least discuss how this assumption affects the results.

The sensitivity analysis section feels a bit thin. You could improve this by explaining the range used for each variable and maybe adding a tornado diagram to show which factors influence the outcome most.

There's no mention of ethical approval or waiver. Even though it’s a modeling study, this should be clarified in the manuscript.

The underlying data and model files haven’t been made publicly available. Please consider sharing them in a repository so others can replicate or build on your work.

The discussion would benefit from addressing real-world implementation challenges -things like infrastructure, funding, or public awareness that affect whether screening programs can actually be rolled out.

With these improvements, your paper could be much stronger and more useful for decision-makers and researchers alike.

Reviewer #2: If the primary objective of the manuscript is a cost-effectiveness analysis, it remains unclear why the authors extend their conclusions to the definition of screening guidelines, particularly with respect to starting age and screening interval.

The statement “Currently, there is no national breast cancer mammography screening program in Nigeria; hence, no national screening guidelines” (lines 58–59) is conceptually problematic. Guidelines do not necessarily follow the existence of a screening program. They may either be developed based on robust evidence tailored to a specific national context or be adapted from existing international guidance. In this manuscript, the authors themselves cite international recommendations (e.g. IARC) that, at a global level, recognize biennial mammography starting at age 50 for women at average risk (references 62–63). The rationale for departing from these established recommendations is not sufficiently justified.

The authors state that evaluating the cost-effectiveness of a national mammography screening program is crucial to ensure accessibility and that a modelling approach is appropriate given the lack of feasibility of trial-based evaluations in Nigeria. While this argument supports the use of modelling, it does not adequately explain how the authors arrive at a recommendation of annual mammography starting at age 40, which is not aligned with IARC recommendations. A cost-effectiveness model alone cannot replace the evidentiary basis required to define screening age and interval.

In addition, the model relies on DALYs as the primary outcome. It is generally accepted that cancer screening programs should primarily be evaluated on mortality reduction, as earlier diagnosis without a demonstrated effect on mortality is often considered a key argument against screening adoption. The use of DALYs may therefore limit the ability of the model to inform screening policy decisions, particularly with regard to overdiagnosis and overtreatment.

A further concern relates to the assumption (line 129) that all breast cancer patients undergo prompt and adequate treatment. This assumption appears inconsistent with the authors’ own introductory statement (line 55) highlighting that early diagnosis of breast cancer in Nigeria faces multiple challenges, resulting in late presentation. The implementation of a screening program presupposes the availability of timely and effective diagnostic and treatment pathways, which are not clearly demonstrated in the manuscript.

It is therefore difficult to understand how the cost-effectiveness model can robustly support conclusions such as those presented in lines 334 and 341, where mammography screening is described as cost-effective from the healthcare payer’s perspective and annual screening is identified as the optimal interval. These conclusions appear to go beyond what can reasonably be inferred from the modelling framework.

The authors also state that “the potential to reduce breast cancer mortality depends on increasing mammography screening rates and facilitating early-stage detection” (line 366). This assertion remains debated in the literature (see, for example, Autier et al., Evaluation of screening mammography effectiveness: The IARC recommendations of 2015 need revision, European Journal of Cancer, 2025). In this context, it is difficult to justify recommendations favoring mammography screening for Nigerian women aged 40–69 based on a DALY-based cost-effectiveness analysis that is not consistent with IARC guidance.

Finally, the statement regarding “current evidence on the increasing incidence of breast cancer in women aged 40–49” (lines 389–391) requires clearer justification and stronger referencing. At present, much of the observed increase in incidence in this age group is attributable to the introduction of screening itself, particularly in settings where screening was previously absent or has been recently extended to younger women. This phenomenon does not provide evidence of screening effectiveness and is widely regarded as a manifestation of overdiagnosis, with consequent overtreatment.

My comments focus on the coherence between the model assumptions, the interpretation of the results, and their implications for screening policy and clinical practice but as public health expert I may not be fully qualified to assess all the methodological nuances of the modelling approach itself. In my opinion in any case, the manuscript presents substantial conceptual and methodological limitations that in its current form prevent it from adequately supporting its main conclusions .

Reviewer #3: The manuscript evaluates the cost-effectiveness of annual versus biennial mammography for Nigerian women starting at age 40 using a lifetime Markov model with DALYs and 5% discounting. The question is policy-relevant and aligned with Nigeria’s cancer-control priorities, and the overall analytic approach is reasonable for screening evaluation. The authors also make a good-faith attempt to characterize uncertainty through sensitivity analyses and acceptability outputs, and they draw on Nigerian inputs where available, which improves local relevance.

However, several issues need to be corrected or more rigorously handled before the findings can be interpreted confidently for policy. First, the stated mortality structure—namely that only stage IV patients can die from breast cancer—is not clinically plausible and is likely to distort life-years/DALYs and therefore the ICER; moreover, the manuscript appears internally inconsistent on this point given the inclusion of stage-specific fatality parameters, so the authors should clarify what was actually implemented. In either case, the model should be revised to a realistic stage-specific mortality framework (or calibrated to observed survival), and the impact of alternative mortality structures should be explored in scenario analyses. Second, the model presumes that all breast cancer patients receive prompt and adequate treatment, which does not reflect real-world variability in access, timeliness, and completion of care in Nigeria and may overstate screening benefits. The analysis should incorporate scenarios with constrained access, treatment delays, and incomplete treatment (including stage-specific treatment coverage if possible), and clearly report how these change outcomes and conclusions. Third, the model description should explicitly specify how screening uptake, adherence across rounds, diagnostic follow-up completion after abnormal screens, and loss-to-follow-up are represented in the base case. If any of these are not modeled or are implicitly assumed to be perfect (e.g., 100% uptake/adherence/follow-up), that should be stated plainly and tested with plausible ranges because these parameters strongly influence both effectiveness and cost-effectiveness—particularly in settings where participation and follow-up can be substantial bottlenecks. Fourth, the exclusion of implementation and other non-medical program costs (e.g., outreach, invitation/recall systems, administration, quality assurance, patient navigation, and program management) will tend to bias results in favor of screening. Even if Nigeria-specific estimates are limited, the authors should include plausible ranges informed by comparable programs and conduct scenario analyses to assess whether conclusions remain robust once these costs are incorporated. Finally, the current data/model availability statement is unlikely to enable full replication of a lifetime decision model. Sharing the model file/code and providing a complete parameter table (including all transition probabilities, screening and follow-up pathways, treatment assumptions, the full costing build-up, and sources) as supplementary material would substantially strengthen transparency, reproducibility, and confidence in the results.

Reviewer #4: It would be appropriate to highlight more clearly the originality of the study by emphasizing how it differs from and adds to existing studies.

In addition, it would be appropriate to describe more clearly—also through a dedicated paragraph—the characteristics of the Nigerian healthcare system and of mammography screening.

Finally, it would also be appropriate to highlight potential future directions for the research.

**********

6. PLOS authors have the option to publish the peer review history of their article (what does this mean?). If published, this will include your full peer review and any attached files.

If you choose “no”, your identity will remain anonymous but your review may still be made public.

**Do you want your identity to be public for this peer review?**  For information about this choice, including consent withdrawal, please see our Privacy Policy.

Reviewer #1: No

Reviewer #2: No

Reviewer #3: No

Reviewer #4: No

**********

[NOTE: If reviewer comments were submitted as an attachment file, they will be attached to this email and accessible via the submission site. Please log into your account, locate the manuscript record, and check for the action link "View Attachments". If this link does not appear, there are no attachment files.]

To ensure your figures meet our technical requirements, please review our figure guidelines: https://journals.plos.org/plosone/s/figures

You may also use PLOS’s free figure tool, NAAS, to help you prepare publication quality figures: https://journals.plos.org/plosone/s/figures#loc-tools-for-figure-preparation.

NAAS will assess whether your figures meet our technical requirements by comparing each figure against our figure specifications.

# Author comment: Author response to Decision Letter 1

**DOI:** 10.1371/journal.pone.0351492.r002

2 Mar 2026

Editor

1. Please ensure that your manuscript meets PLOS ONE's style requirements, including those for file naming. The PLOS ONE style templates can be found at https://journals.plos.org/plosone/s/file?id=wjVg/PLOSOne_formatting_sample_main_body.pdf and https://journals.plos.org/plosone/s/file?id=ba62/PLOSOne_formatting_sample_title_authors_affiliations.pdf.

Thank you for taking your time to review our manuscript.

We have formatted the manuscript to meet PLOS One’s style requirement, particularly those for file naming.

2. Please note that PLOS One has specific guidelines on code sharing for submissions in which author-generated code underpins the findings in the manuscript. In these cases, we expect all author-generated code to be made available without restrictions upon publication of the work. Please review our guidelines at https://journals.plos.org/plosone/s/materials-and-software-sharing#loc-sharing-code and ensure that your code is shared in a way that follows best practice and facilitates reproducibility and reuse.

We have deposited our data in Mendeley Data at https://data.mendeley.com/datasets/5k9zwnz4yb/1.

3. We noticed you have some minor occurrence of overlapping text with the following previous publication(s), which needs to be addressed:

https://bmjpublichealth.bmj.com/content/2/2/e001356.

https://journals.plos.org/plosntds/article?id=10.1371%2Fjournal.pntd.0006124

In your revision ensure you cite all your sources (including your own works), and quote or rephrase any duplicated text outside the methods section. Further consideration is dependent on these concerns being addressed.

We have addressed the occurrence of overlapping texts and cited all our sources, especially my works.

4. Thank you for stating in your Funding Statement:

“This study was partly funded by the Royal Society of Tropical Medicine and Hygiene (RSTMH) in partnership with the National Institute for Health Research (NIHR), 2021 Small Grants Programme. The funders had no role in study design, data collection and analysis, decision to publish, or preparation of the manuscript.”

Please provide an amended statement that declares *all* the funding or sources of support (whether external or internal to your organization) received during this study, as detailed online in our guide for authors at http://journals.plos.org/plosone/s/submit-now. Please also include the statement “There was no additional external funding received for this study.” in your updated Funding Statement.

Please include your amended Funding Statement within your cover letter. We will change the online submission form on your behalf.

The funding statement was amended as follows, “This study was mostly funded by the Royal Society of Tropical Medicine and Hygiene (RSTMH) in partnership with the National Institute for Health Research (NIHR), 2021 Small Grants Programme. The corresponding author, IJN, funded some parts of the study. The external funders had no role in study design, data collection and analysis, decision to publish, or preparation of the manuscript.”

5. Please provide a complete Data Availability Statement in the submission form, ensuring you include all necessary access information or a reason for why you are unable to make your data freely accessible. If your research concerns only data provided within your submission, please write "All data are in the manuscript and/or supporting information files" as your Data Availability Statement.

The data availability statement was re-written as follows, “All data and code are available in the Mendeley Data at https://data.mendeley.com/datasets/5k9zwnz4yb/1(DOI: 10.17632/5k9zwnz4yb.1).”

6. Please be informed that funding information should not appear in the Acknowledgments section or other areas of your manuscript. We will only publish funding information present in the Funding Statement section of the online submission form. Please remove any funding-related text from the manuscript.

The funding statement has been removed from the manuscript.

7. If the reviewer comments include a recommendation to cite specific previously published works, please review and evaluate these publications to determine whether they are relevant and should be cited. There is no requirement to cite these works unless the editor has indicated otherwise.

Not applicable

Reviewer 1

1. The Markov model you’ve used is appropriate, but several of the input values—like transition probabilities and utility scores—are taken from studies outside Nigeria or are outdated. It would help to either source more locally relevant data or explain clearly why these were used.

Thank you for taking your time to review our manuscript.

We used country-specific data were available and used data for countries with similar health characteristics when not available. This was acknowledged in the study limitation. However, we explored the uncertainties in the data utilized using the sensitivity analysis.

2. Assuming 100 percent screening participation is unrealistic in the Nigerian context. Please try modeling lower compliance rates or at least discuss how this assumption affects the results.

The reviewer is right. As 100 percent screening participation is unrealistic in Nigeria, we have now modeled a 50 percent participation rate and updated the manuscript accordingly.

3. The sensitivity analysis section feels a bit thin. You could improve this by explaining the range used for each variable and maybe adding a tornado diagram to show which factors influence the outcome most.

Thank you for this observation. The sensitivity analysis section includes a tornado diagram that captured the ranges used for the various variables. However, they were not included in the manuscript as stipulated by the journal guidelines, rather it was updated as a separate file. Also, the distribution of the variables was already discussed under the analysis section of the methodology and shown in Table 1.

4. There's no mention of ethical approval or waiver. Even though it’s a modeling study, this should be clarified in the manuscript.

We followed the journal guidelines on the submission for a study not involving human or animal subjects. Hence, we have included that “an ethics statement was not required for this work” in the method section.

5. The underlying data and model files haven’t been made publicly available. Please consider sharing them in a repository so others can replicate or build on your work.

We have deposited our data set in the Mendeley Data and updated the manuscript accordingly.

6. The discussion would benefit from addressing real-world implementation challenges -things like infrastructure, funding, or public awareness that affect whether screening programs can actually be rolled out.

Thank you for this comment. We have included solutions to real-world implementation challenges that could emerge (see page 22).

Reviewer 2

1. If the primary objective of the manuscript is a cost-effectiveness analysis, it remains unclear why the authors extend their conclusions to the definition of screening guidelines, particularly with respect to starting age and screening interval.

We have now revised the aim and conclusion for clarity. The study aimed to assess the cost-effective and optimal screening interval of mammography screening.

2. The statement “Currently, there is no national breast cancer mammography screening program in Nigeria; hence, no national screening guidelines” (lines 58–59) is conceptually problematic. Guidelines do not necessarily follow the existence of a screening program. They may either be developed based on robust evidence tailored to a specific national context or be adapted from existing international guidance. In this manuscript, the authors themselves cite international recommendations (e.g. IARC) that, at a global level, recognize biennial mammography starting at age 50 for women at average risk (references 62–63). The rationale for departing from these established recommendations is not sufficiently justified.

This reviewer is right, and we have now revised our statement in line with the reviewer’s suggestion. We acknowledge that although there is currently no screening program in the country, clinicians depend on international guidelines to screen their patients, which may be the best option for the patients. Therefore, we have now deleted the claim that there is “no screening guideline”.

3. The authors state that evaluating the cost-effectiveness of a national mammography screening program is crucial to ensure accessibility and that a modelling approach is appropriate given the lack of feasibility of trial-based evaluations in Nigeria. While this argument supports the use of modelling, it does not adequately explain how the authors arrive at a recommendation of annual mammography starting at age 40, which is not aligned with IARC recommendations. A cost-effectiveness model alone cannot replace the evidentiary basis required to define screening age and interval.

We thank the reviewer for this important clarification and agree that our original wording overstated the implications of the cost-effectiveness analysis. Randomised controlled trial-based economic evaluations remains the gold standard for establishing screening age and interval, and a modelling study alone cannot substitute for the evidentiary foundation required to define national screening policy. We also acknowledge that our cost-effectiveness analysis does not, in itself, ensure accessibility as previously claimed, but rather provides supportive economic evidence to inform decision making alongside considerations of feasibility, equity, and health system capacity.

In our model, age 40 and annual screening was included as pre-specified exploratory scenarios to allow comparison across plausible screening strategies, rather than to recommend an optimal national policy. Although the model identified annual screening from age 40 as cost-effective under certain assumptions, this finding does not constitute Level 1 evidence and should not be interpreted as a policy recommendation, particularly given its divergence from IARC guidance.

We have now revised the manuscript to clarify that the purpose of the modelling analysis is to assess the value for money of alternative screening strategies, not to define the appropriate starting age or screening interval. The revised text now reads:

“…it is crucial to evaluate the value for money of a national mammography screening in Nigeria to support decision making. While a randomised controlled trial-based economic evaluation would be ideal, such studies may not currently be feasible. In this context, a robust modelling approach can provide a feasible complementary source of evidence to compare alternative screening scenarios.”

4. In addition, the model relies on DALYs as the primary outcome. It is generally accepted that cancer screening programs should primarily be evaluated on mortality reduction, as earlier diagnosis without a demonstrated effect on mortality is often considered a key argument against screening adoption. The use of DALYs may therefore limit the ability of the model to inform screening policy decisions, particularly with regard to overdiagnosis and overtreatment.

We agree on the importance of mortality reduction as an outcome for cancer screening. We have now provided this evidence as a secondary outcome. No intervention = 6,447 deaths (623 deaths per 100,000 population); Annual = 2,557 death (247 deaths per 100,000 population); Biennial = 3,616 deaths (349 deaths per 100,000 population). This has been included in the manuscript.

5. A further concern relates to the assumption (line 129) that all breast cancer patients undergo prompt and adequate treatment. This assumption appears inconsistent with the authors’ own introductory statement (line 55) highlighting that early diagnosis of breast cancer in Nigeria faces multiple challenges, resulting in late presentation. The implementation of a screening program presupposes the availability of timely and effective diagnostic and treatment pathways, which are not clearly demonstrated in the manuscript.

The model presumed that all breast cancer patients underwent prompt and adequate treatment. This was supposedly so, because the model assumed an effective screening approach in place, which will ensure prompt and adequate screening. Given that this may not be absolutely feasible in a setting like Nigeria, we have discussed this a limitation in our model in the Discussion section.

6. It is therefore difficult to understand how the cost-effectiveness model can robustly support conclusions such as those presented in lines 334 and 341, where mammography screening is described as cost-effective from the healthcare payer’s perspective and annual screening is identified as the optimal interval. These conclusions appear to go beyond what can reasonably be inferred from the modelling framework.

Thank you for this important comment. We agree that the original wording overstated the strength of the conclusions that can be drawn from the modelling framework. The cost-effectiveness model was designed to compare alternative screening scenarios under a defined set of assumptions, rather than to establish definitive screening intervals or policy recommendations. We have therefore revised the relevant sections to clarify that, while mammography screening scenarios were cost-effective from the healthcare payer’s perspective within the model, the identification of annual screening as the most favorable option reflects model-specific results rather than a robust or prescriptive conclusion. These findings should be interpreted as exploratory and hypothesis-generating. The revised text (page 20) now emphasizes that the analysis provides comparative economic insights to inform future research and policy discussions, rather than definitive guidance on screening intervals.

7. The authors also state that “the potential to reduce breast cancer mortality depends on increasing mammography screening rates and facilitating early-stage detection” (line 366). This assertion remains debated in the literature (see, for example, Autier et al., Evaluation of screening mammography effectiveness: The IARC recommendations of 2015 need revision, European Journal of Cancer, 2025). In this context, it is difficult to justify recommendations favoring mammography screening for Nigerian women aged 40–69 based on a DALY-based cost-effectiveness analysis that is not consistent with IARC guidance.

We agree with the reviewer. We have now revised the statement as shown below:

“The potential to reduce breast cancer mortality depends on the frequency of mammography screening rates and facilitating early-stage detection”.

8. Finally, the statement regarding “current evidence on the increasing incidence of breast cancer in women aged 40–49” (lines 389–391) requires clearer justification and stronger referencing. At present, much of the observed increase in incidence in this age group is attributable to the introduction of screening itself, particularly in settings where screening was previously absent or has been recently extended to younger women. This phenomenon does not provide evidence of screening effectiveness and is widely regarded as a manifestation of overdiagnosis, with consequent overtreatment.

We agree that Increase in incidence in this age group is due to the introduction of screening itself. We intended to write on the relative reduction in breast cancer mortality with the start of screening in this age group. With strong references, we have now revised the statement as follows: “This is due to the increasing evidence of relative reduction in breast cancer mortality with commencement of mammography screening at this age”

Reviewer 3

The manuscript evaluates the cost-effectiveness of annual versus biennial mammography for Nigerian women starting at age 40 using a lifetime Markov model with DALYs and 5% discounting. The question is policy-relevant and aligned with Nigeria’s cancer-control priorities, and the overall analytic approach is reasonable for screening evaluation. The authors also make a good-faith attempt to characterize uncertainty through sensitivity analyses and acceptability outputs, and they draw on Nigerian in

Attachment Submitted filename: Response to Reviewers.docx

# Peer review: Decision Letter 1

**DOI:** 10.1371/journal.pone.0351492.r003

**Authors:** Daniele Ugo Tari

25 Mar 2026

PONE-D-25-51694R1

Cost-effectiveness analysis of mammography screening for early detection of breast cancer in Nigeria

PLOS One

Dear Dr. Nduka,

Thank you for submitting your manuscript to PLOS ONE. After careful consideration, we feel that it has merit but does not fully meet PLOS ONE’s publication criteria as it currently stands. Therefore, we invite you to submit a revised version of the manuscript that addresses the points raised during the review process.

Please submit your revised manuscript by May 09 2026 11:59PM. If you will need more time than this to complete your revisions, please reply to this message or contact the journal office at plosone@plos.org. When you're ready to submit your revision, log on to https://www.editorialmanager.com/pone/ and select the 'Submissions Needing Revision' folder to locate your manuscript file.

Please include the following items when submitting your revised manuscript:

- A letter that responds to each point raised by the academic editor and reviewer(s). You should upload this letter as a separate file labeled 'Response to Reviewers'.
- A marked-up copy of your manuscript that highlights changes made to the original version. You should upload this as a separate file labeled 'Revised Manuscript with Track Changes'.
- An unmarked version of your revised paper without tracked changes. You should upload this as a separate file labeled 'Manuscript'.

If you would like to make changes to your financial disclosure, please include your updated statement in your cover letter. Guidelines for resubmitting your figure files are available below the reviewer comments at the end of this letter.

If applicable, we recommend that you deposit your laboratory protocols in protocols.io to enhance the reproducibility of your results. Protocols.io assigns your protocol its own identifier (DOI) so that it can be cited independently in the future. For instructions see: https://journals.plos.org/plosone/s/submission-guidelines#loc-laboratory-protocols. Additionally, PLOS ONE offers an option for publishing peer-reviewed Lab Protocol articles, which describe protocols hosted on protocols.io. Read more information on sharing protocols at https://plos.org/protocols?utm_medium=editorial-email&utm_source=authorletters&utm_campaign=protocols.

As the corresponding author, your ORCID iD is verified in the submission system and will appear in the published article. PLOS supports the use of ORCID, and we encourage all coauthors to register for an ORCID iD and use it as well. Please encourage your coauthors to verify their ORCID iD within the submission system before final acceptance, as unverified ORCID iDs will not appear in the published article. *Only*  the individual author can complete the verification step; PLOS staff *cannot*  verify ORCID iDs on behalf of authors.

We look forward to receiving your revised manuscript.

Kind regards,

Daniele Ugo Tari, M.D.

Academic Editor

PLOS One

Journal Requirements:

1. If the reviewer comments include a recommendation to cite specific previously published works, please review and evaluate these publications to determine whether they are relevant and should be cited. There is no requirement to cite these works unless the editor has indicated otherwise.

2. Please review your reference list to ensure that it is complete and correct. If you have cited papers that have been retracted, please include the rationale for doing so in the manuscript text, or remove these references and replace them with relevant current references. Any changes to the reference list should be mentioned in the rebuttal letter that accompanies your revised manuscript. If you need to cite a retracted article, indicate the article’s retracted status in the References list and also include a citation and full reference for the retraction notice.

Additional Editor Comments:

Dear Authors,

The paper has been widely revised according to the reviewers' suggestions.

Nevertheless, there are a few observations from reviewer n. 2 requiring revision.

Sincerely,

[Note: HTML markup is below. Please do not edit.]

Reviewer's Responses to Questions

**Comments to the Author**

1. If the authors have adequately addressed your comments raised in a previous round of review and you feel that this manuscript is now acceptable for publication, you may indicate that here to bypass the “Comments to the Author” section, enter your conflict of interest statement in the “Confidential to Editor” section, and submit your "Accept" recommendation.

Reviewer #2: All comments have been addressed

Reviewer #4: All comments have been addressed

**********

2. Is the manuscript technically sound, and do the data support the conclusions?

The manuscript must describe a technically sound piece of scientific research with data that supports the conclusions. Experiments must have been conducted rigorously, with appropriate controls, replication, and sample sizes. The conclusions must be drawn appropriately based on the data presented.

Reviewer #2: (No Response)

Reviewer #4: Yes

**********

3. Has the statistical analysis been performed appropriately and rigorously?

Reviewer #2: I Don't Know

Reviewer #4: Yes

**********

4. Have the authors made all data underlying the findings in their manuscript fully available?

The PLOS Data policy requires authors to make all data underlying the findings described in their manuscript fully available without restriction, with rare exception (please refer to the Data Availability Statement in the manuscript PDF file). The data should be provided as part of the manuscript or its supporting information, or deposited to a public repository. For example, in addition to summary statistics, the data points behind means, medians and variance measures should be available. If there are restrictions on publicly sharing data—e.g. participant privacy or use of data from a third party—those must be specified.

Reviewer #2: Yes

Reviewer #4: Yes

**********

5. Is the manuscript presented in an intelligible fashion and written in standard English?

PLOS ONE does not copyedit accepted manuscripts, so the language in submitted articles must be clear, correct, and unambiguous. Any typographical or grammatical errors should be corrected at revision, so please note any specific errors here.

Reviewer #2: Yes

Reviewer #4: Yes

**********

6. Review Comments to the Author

Please use the space provided to explain your answers to the questions above. You may also include additional comments for the author, including concerns about dual publication, research ethics, or publication ethics. (Please upload your review as an attachment if it exceeds 20,000 characters)

Reviewer #2: Overall, the revisions introduced by the authors respond satisfactorily to the previous comments and represent a meaningful improvement of the manuscript. It is understandable that the modelling approach focuses on the 40–49 age group, which currently does not comply with international recommendations that generally indicate the start of screening at age 50. This limitation is correctly acknowledged in the manuscript but the issue might be further contextualized not only by recalling references 60–63, which, in my opinion, do not have the authority to be considered a valid alternative to IARC recommendations, but also by considering perhaps the demographic characteristics of Nigeria, whose relatively young median age may partly support the authors’ focus on the 40–49 age group. Nevertheless, the question of the transferability of a purely economic modelling analysis to a real-world implementation context remains open, particularly when the target age group and screening frequency differ from those universally recommended by IARC. In this respect, the practical implementation considerations discussed on page 22 add value to the manuscript. On the one hand, they outline possible pathways for translating the theoretical modelling results into real-world practice; on the other hand, they appropriately acknowledge the limitations inherent to a theoretical economic model when informing screening policy decisions.

Reviewer #4: (No Response)

**********

7. PLOS authors have the option to publish the peer review history of their article (what does this mean?). If published, this will include your full peer review and any attached files.

If you choose “no”, your identity will remain anonymous but your review may still be made public.

**Do you want your identity to be public for this peer review?**  For information about this choice, including consent withdrawal, please see our Privacy Policy.

Reviewer #2: **Yes:**  Francesco Gongolo

Reviewer #4: No

**********

[NOTE: If reviewer comments were submitted as an attachment file, they will be attached to this email and accessible via the submission site. Please log into your account, locate the manuscript record, and check for the action link "View Attachments". If this link does not appear, there are no attachment files.]

To ensure your figures meet our technical requirements, please review our figure guidelines: https://journals.plos.org/plosone/s/figures

You may also use PLOS’s free figure tool, NAAS, to help you prepare publication quality figures: https://journals.plos.org/plosone/s/figures#loc-tools-for-figure-preparation.

NAAS will assess whether your figures meet our technical requirements by comparing each figure against our figure specifications.

# Author comment: Author response to Decision Letter 2

**DOI:** 10.1371/journal.pone.0351492.r004

9 May 2026

Reviewer #2

Thank you once more for reviewing our manuscript. We deeply appreciate your suggestion about Nigeria’s demographics.

We never intended to present references 60-63 as equivalent alternatives to IARC’s guidance as it remains an international consensus. The references are evidence supporting context-specific flexibility. We have now revised the manuscript (page 24) and it reads:

“While the International Agency for Research on Cancer (IARC) guidelines recommend screening initiation at age 50, emerging regional evidence and national burden data have informed context-adapted approaches in low- and middle-income settings. There are increasing evidence of relative reduction in breast cancer mortality with commencement of mammography screening at this age [60–63], and early breast cancer cases in Africa; a meta-analysis showed that 58% of diagnosed patients were less than 50 years [64]. Also, Nigeria’s relatively young median age (18-19 years) and the substantial proportion of the adult population in the 40-49 age bracket provided a demographic rationale for modeling this age group [65], though it falls outside IARC’s standard recommendation.”

Thank you for appreciating the value of the practical implementation considerations. Regarding the age group and screening frequency deviating from IARC guidance, we have included the following in the manuscript (page 22):

“With the modeled age group and screening interval deviating from the International Agency for Research on Cancer (IARC) recommendations, there would be a need for policy makers to deliberate on aligning with or justifying deviations from the guidance.”

Attachment Submitted filename: Response_to_Reviewers_auresp_2.docx

# Editor report: Decision Letter 2

**DOI:** 10.1371/journal.pone.0351492.r005

**Authors:** Daniele Ugo Tari

29 May 2026

Cost-effectiveness analysis of mammography screening for early detection of breast cancer in Nigeria

PONE-D-25-51694R2

Dear Dr. Nduka,

We’re pleased to inform you that your manuscript has been judged scientifically suitable for publication and will be formally accepted for publication once it meets all outstanding technical requirements.

Within one week, you’ll receive an e-mail detailing the required amendments. When these have been addressed, you’ll receive a formal acceptance letter and your manuscript will be scheduled for publication.

An invoice will be generated when your article is formally accepted. Please note, if your institution has a publishing partnership with PLOS and your article meets the relevant criteria, all or part of your publication costs will be covered. Please make sure your user information is up-to-date by logging into Editorial Manager at Editorial Manager® and clicking the ‘Update My Information' link at the top of the page. For questions related to billing, please contact billing support.

If your institution or institutions have a press office, please notify them about your upcoming paper to help maximize its impact. If they’ll be preparing press materials, please inform our press team as soon as possible -- no later than 48 hours after receiving the formal acceptance. Your manuscript will remain under strict press embargo until 2 pm Eastern Time on the date of publication. For more information, please contact onepress@plos.org.

Kind regards,

Daniele Ugo Tari, M.D.

Academic Editor

PLOS One

Additional Editor Comments (optional):

Dear Authors,

All comments have been addressed.

The paper has significantly improved and consequently, it can be accepted for publication.

Sincerely,

Reviewers' comments:

# Editor report: Acceptance letter

**DOI:** 10.1371/journal.pone.0351492.r006

**Authors:** Daniele Ugo Tari

PONE-D-25-51694R2

PLOS One

Dear Dr. Nduka,

I'm pleased to inform you that your manuscript has been deemed suitable for publication in PLOS One. Congratulations! Your manuscript is now being handed over to our production team.

At this stage, our production department will prepare your paper for publication. This includes ensuring the following:

* All references, tables, and figures are properly cited

* All relevant supporting information is included in the manuscript submission,

* There are no issues that prevent the paper from being properly typeset

You will receive further instructions from the production team, including instructions on how to review your proof when it is ready. Please keep in mind that we are working through a large volume of accepted articles, so please give us a few days to review your paper and let you know the next and final steps.

Lastly, if your institution or institutions have a press office, please let them know about your upcoming paper now to help maximize its impact. If they'll be preparing press materials, please inform our press team within the next 48 hours. Your manuscript will remain under strict press embargo until 2 pm Eastern Time on the date of publication. For more information, please contact onepress@plos.org.

You will receive an invoice from PLOS for your publication fee after your manuscript has reached the completed accept phase. If you receive an email requesting payment before acceptance or for any other service, this may be a phishing scheme. Learn how to identify phishing emails and protect your accounts at https://explore.plos.org/phishing.

If we can help with anything else, please email us at customercare@plos.org.

Thank you for submitting your work to PLOS ONE and supporting open access.

Kind regards,

PLOS ONE Editorial Office Staff

on behalf of

Dr. Daniele Ugo Tari

Academic Editor

PLOS One
